# Sarcopenia Severity and the Accumulation of Geriatric Syndromes Among Older Adults: A Cross-Sectional Study from Vietnam

**DOI:** 10.3390/geriatrics11030051

**Published:** 2026-04-23

**Authors:** Huong Thi Thu Nguyen, Vasi Naganathan, Thanh Xuan Nguyen, Tam Ngoc Nguyen, Thu Thi Hoai Nguyen, Huyen Thi Thanh Vu, Anh Lan Nguyen, Vien Chi Le, Narelle Warren, Hoa Lan Nguyen, Robert J. Goldberg, Anh Trung Nguyen

**Affiliations:** 1 Department of Geriatrics, Hanoi Medical University, Hanoi 11500, Vietnam; thuhuonglk@hmu.edu.vn (H.T.T.N.); xuanthanhbmlk@hmu.edu.vn (T.X.N.); ngoctam@hmu.edu.vn (T.N.N.); nththu.bvlk2@gmail.com (T.T.H.N.); vuthanhhuyen11@hmu.edu.vn (H.T.T.V.); min_moon85@yahoo.com (A.L.N.); 2 Scientific Research Department, National Geriatric Hospital, Hanoi 11500, Vietnam; 3 Department of Geriatric Medicine, Centre for Education and Research on Ageing (CERA), Concord Hospital, Sydney, NSW 2139, Australia; vasi.naganathan@sydney.edu.au; 4 Faculty of Medicine and Health, University of Sydney, Sydney, NSW 2006, Australia; 5 Stroke Department, 108 Military Central Hospital, Hanoi 11600, Vietnam; dr.chivien.bv108@gmail.com; 6 School of Social Sciences, Monash University, Clayton, VIC 3800, Australia; narelle.warren@monash.edu; 7 Department of Population and Quantitative Health Sciences, University of Massachusetts Chan Medical School, Worcester, MA 01655, USA; hoa.nguyen@umassmed.edu (H.L.N.); robert.goldberg@umassmed.edu (R.J.G.)

**Keywords:** sarcopenia, severe sarcopenia, geriatric syndromes, comprehensive geriatric assessment, Vietnam

## Abstract

**Background/Objectives:** Sarcopenia frequently coexists with other geriatric syndromes, and its severity may influence their clinical manifestation. This study examines the prevalence of geriatric syndromes in older adults with non-severe and severe sarcopenia and explores the associations between sarcopenia severity and individual geriatric syndromes in Vietnam. **Methods**: A cross-sectional study was conducted among 726 older outpatients with sarcopenia. Non-severe and severe sarcopenia were diagnosed according to the Asian Working Group for Sarcopenia algorithm. Fifteen geriatric conditions spanning physical and psychological health, functional status, and social circumstances were assessed using components of the Comprehensive Geriatric Assessment. Logistic regression models were used to examine associations between sarcopenia severity and geriatric syndromes. **Results**: A total of 726 older patients with sarcopenia (mean age 74.4 years, 77.4% females) were included, of whom 53.4% had severe sarcopenia. A significantly higher prevalence of geriatric syndromes was observed in patients with severe compared with non-severe sarcopenia, including sleep disturbances (79.4% vs. 67.5%), frailty (71.4% vs. 54.7%), malnourishment/risk of malnutrition (61.9% vs. 50.0%), depression (54.9% vs. 34.9%), polypharmacy (49.5% vs. 42.0%), impairment in activities of daily living (52.8% vs. 32.5%), and impairment in instrumental activities of daily living (58.2% vs. 39.3%). After adjustment for potential confounders, severe sarcopenia remained associated with sleep disturbance (adjusted OR 1.49, 95%CI 1.02–2.18, *p* = 0.046), depression (adjusted OR 1.90, 95%CI 1.36–2.66, *p* < 0.001), and mobility impairment (adjusted OR 3.01, 95%CI 2.12–4.27, *p* < 0.001). **Conclusions**: Older Vietnamese adults with sarcopenia had a high burden of geriatric syndromes, particularly among those with severe disease. Severe sarcopenia was independently associated with sleep disturbance, depression, and impaired mobility—clinically relevant and potentially modifiable conditions. These findings highlight the importance of evaluating sarcopenia within a broader geriatric framework and may inform early identification and prioritization of coexisting geriatric syndromes, especially in resource-limited settings.

## 1. Introduction

Sarcopenia, a common condition in older adults, is characterized by age-related loss of skeletal muscle mass and function and is associated with multiple adverse health outcomes in this population [1,2,3]. In Asia, the frequency of sarcopenia has ranged from 10 to 30% in the community [1,4] to upwards of 50% among hospitalized patients [5,6]. Sarcopenia has been linked to frailty, increased risks of cardiovascular and respiratory disease, and higher all-cause mortality [7,8]. Severe sarcopenia, which is characterized by significant reductions in muscle mass, muscle strength, and physical performance, affects approximately 2% to 9% of older adults [9]. This advanced stage has been associated with poor cognitive function, an increased risk of falls, adverse postoperative outcomes, and higher mortality [10,11,12].

Previous studies, largely from high-income countries, have documented a high prevalence of individual geriatric conditions in adults with sarcopenia, including vision and hearing loss [13], frailty [14], cognitive impairment [15,16], depression [17], and sleep disorders [18,19]. These findings suggest that sarcopenia may identify a population with a higher likelihood of having multiple co-existing geriatric conditions. Although nutrition and exercise remain central to sarcopenia management, understanding the broader health burden associated with sarcopenia—particularly the extent to which this burden increases with disease severity—is essential for guiding clinical decision-making and resource prioritization. One potential implication is that the identification of sarcopenia, especially in its severe stage, may signal the need for a comprehensive geriatric assessment (CGA) of older adults [20,21,22].

As one of the countries with the most rapidly aging populations in the world [23], Vietnam faces considerable challenges in scaling up geriatric services, particularly in the context of a still-developing workforce and healthcare infrastructure. While awareness of sarcopenia is growing in both community and hospital setting in Vietnam [4,6], little is known about how sarcopenia severity relates to the cumulative burden of geriatric syndromes in this setting, and evidence from low- and middle-income countries remains scarce.

The present study aimed to assess the prevalence of several common geriatric syndromes among Vietnamese adults aged 60 years and older with non-severe and severe sarcopenia. We further examined whether the frequency of these age-related conditions differed according to sarcopenia severity and whether there was an association between sarcopenia severity and individual geriatric syndromes. By characterizing the gradient of geriatric syndrome burden across sarcopenia severity levels, our findings provide locally relevant data to guide clinical prioritization and care planning for older adults in resource-limited healthcare settings.

## 2. Materials and Methods

### 2.1. Study Design and Participants

This cross-sectional study was conducted at the internal medicine clinics of the Outpatient Department at the National Geriatric Hospital, Hanoi, Vietnam from June to November 2022. The National Geriatric Hospital is a tertiary referral center providing specialized care for older adults and receives outpatient referrals from primary and secondary healthcare facilities across northern Vietnam. As a result, the study population includes patients from Hanoi and surrounding provinces who were referred for evaluation and management of complex geriatric conditions.

Participants were identified and screened from twelve internal medicine clinics. Inclusion criteria were: (1) age ≥ 60 years; (2) attendance at the outpatient internal medicine clinics of the National Geriatric Hospital during the study period; and (3) diagnosis of sarcopenia according to the Asian Working Group for Sarcopenia (AWGS) 2019 criteria.

**Figure 1:**
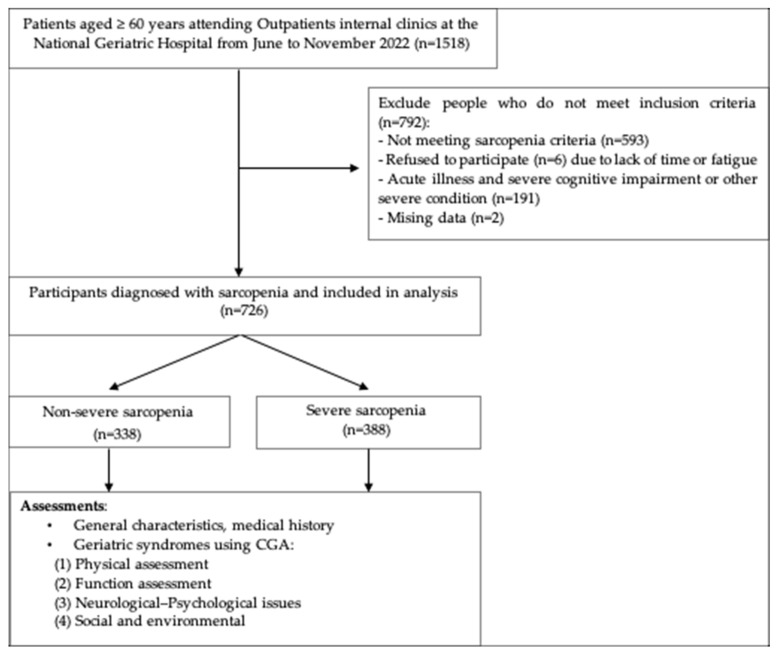
Flowchart of participant selection and inclusion in the study.

Exclusion criteria included: (1) contraindications to the measurement of muscle mass using bioelectrical impedance analysis_BIA (including implanted electronic medical devices (e.g., pacemakers or implantable cardioverter-defibrillators), limb amputation or metallic implants interfering with measurement, and inability to stand independently); (2) acute or unstable medical conditions (e.g., acute infection, acute exacerbation of chronic obstructive pulmonary disease, acute stroke, unstable angina, or acute heart failure); (3) terminal illness (advanced malignancy); or (4) severe cognitive impairment or other severe conditions (e.g., severe liver failure, end-stage renal disease, severe respiratory failure, or severe neurological disorders such as severe Parkinson’s disease or severe post-stroke disability) that could prevent completion of study assessments or affect the reliability of the evaluation results. The participant selection process is illustrated in Figure 1.

### 2.2. Data Collection Activities

Data were collected by a face-to-face assessment using a standardized questionnaire and physical examination, as well as through a review of patients’ medical records by members of the study team. This study was designed to collect the breadth of geriatric syndromes, which would be collected as part of a CGA. At the beginning of the study, study physicians were trained in the administration of the survey questionnaire and how to perform any necessary tests as part of the CGA. A pilot study was conducted among a sample of 20 patients for the purpose of assessing the feasibility of using these tools in this patient group, and we conducted a post-pilot review among the research team to finalize the study tools and protocol.

The following information was collected from eligible and consenting study participants:

Socio-demographic characteristics and medical history: age, gender, education level, living status (with spouse/family or with caregivers/alone), residential area (urban/rural), hospitalizations in the past 12 months, and chronic comorbidities (e.g., hypertension, diabetes, heart failure, stroke, chronic ischemic heart disease, lipid disorders, chronic obstructive pulmonary disease, knee osteoarthritis, and Parkinson’s disease) based on interviews and medical records.

Physical activity: Level of physical activity was evaluated by the International Physical Activity Questionnaire short form (IPAQ-SF) [24] and determined based on the converted energy from the patient’s self-assessment questions (metabolic equivalent task, MET-minutes per week) with further subdivision into 3 levels: low (<600 MET-minutes per week); moderate (600–3000 MET-minutes per week) and high (>3000 MET-minutes per week) [25].

Sarcopenia definition: Sarcopenia was diagnosed according to the Asian Working Group for Sarcopenia 2019 criteria [26] including: (1) The skeletal muscle index (SMI) was evaluated using a Bioelectrical impedance analysis (BIA, Inbody 770). Low muscle mass: SMI < 7.0 kg/m^2^ in males, <5.7 kg/m^2^ in females [26]. (2) Handgrip strength (HGS) was evaluated using a dynamometer (Jamar ^TM^ Hydraulic Hand Dynamometer 5030 J1, USA). Low muscle strength: HGS < 28 kg in males, <18 kg in females [26]. (3) Gait speed was assessed by the 6-m walk test. Low physical performance: gait speed < 1.0 m/s [26].

Participants were classified as having non-severe sarcopenia when the first and second criteria, or when the first and third criteria, were met. Severe sarcopenia was diagnosed when all three criteria were determined to be present.

Geriatric syndromes: Geriatric syndromes were assessed using components of the Comprehensive Geriatric Assessment (CGA). CGA was performed by physicians on the research team. The assessment tools, methods, and diagnostic criteria utilized for our working CGA are shown in Table 1.

### 2.3. Data Analysis

Continuous variables were presented as means and standard deviations (SD) and compared between patients with non-severe sarcopenia and those with severe sarcopenia using *t*-tests. Categorical variables were presented as frequencies and percentages and compared between the two primary comparison groups (severe and non-severe sarcopenia) using the Chi-square test. The normality of continuous variables was assessed using the Shapiro–Wilk test, visual inspection of histograms and Q–Q plots, and examination of skewness and kurtosis values. Skewness and kurtosis values were within acceptable ranges, and visual inspection indicated approximately symmetric distributions without substantial outliers. Given the large sample size in each group (338 and 388 participants), independent samples t-tests were used, as the sampling distribution of the mean approximates normality according to the Central Limit Theorem. Unadjusted and multivariable adjusted logistic regression analysis was performed to examine the association between severe sarcopenia and the various geriatric syndromes: model 1 without adjustment, model 2 with adjustment for age and gender, and model 3 with adjustment for age, gender, level of physical activity, malnutrition, and history of hypertension or diabetes. Covariates were selected a priori based on clinical relevance and the prior literature, demonstrating their associations with both sarcopenia severity and geriatric syndromes. Model goodness of fit was assessed using Nagelkerke R^2^, the Hosmer–Lemeshow test, and classification accuracy. Data were analyzed using SPSS 26.0 software.

## 3. Results

The study sample consisted of 726 older adults with sarcopenia. The mean age of participants was 74.4 ± 8.0 years, and 77.4% were females. More than half of the study population (388 participants, 53.4%) had severe sarcopenia.

### 3.1. Participant Characteristics According to Sarcopenia Severity

Patients with severe sarcopenia were significantly older than those with non-severe sarcopenia. The proportion of patients with one or more hospitalizations in the previous 12 months, the presence of comorbidities (hypertension, diabetes), and low levels of physical activity were significantly higher in the severe sarcopenia group (Table 2).

### 3.2. Prevalence of Geriatric Syndromes According to Sarcopenia Severity

In examining the prevalence of various geriatric syndromes in patients with non-severe and severe sarcopenia, more than one-third of participants in both groups exhibited sleep disorders, frailty, depression, visual impairment, hearing impairment, polypharmacy, and IADL impairment (Table 3). Sleep disorders were the most prevalent condition and were significantly more common among patients with severe sarcopenia compared with those with non-severe sarcopenia (79.4% vs. 67.5%). Several additional conditions were significantly more frequent in the severe sarcopenia group, including higher Charlson comorbidity index, polypharmacy, malnutrition/risk of malnutrition, urinary incontinence, frailty, ADL impairment, IADL impairment, mobility impairment, cognitive impairment, depression, sleep disturbances, and social isolation (Table 3). The proportion of participants with more than five concurrent geriatric syndromes was greater among those with severe sarcopenia compared with non-severe sarcopenia (68.6% vs. 47.9%).

### 3.3. Association Between Severe Sarcopenia and Geriatric Syndromes

In unadjusted analyses, severe sarcopenia was significantly associated with an increased odds of being frail, ADL, IADL, or mobility impairment, as well as sleep disturbance, polypharmacy, urinary incontinence, cognitive impairment, depression, or social isolation (Table 4). After adjustment for age and gender, severe sarcopenia remained significantly associated with frailty, ADL, IADL and mobility impairment, sleep disturbances, cognitive impairment, depression, and social isolation. In the fully adjusted multivariable logistic regression models—accounting for age, gender, physical activity, malnutrition, hypertension, and diabetes—the associations with sleep disturbances, depression, and mobility impairment remained statistically significant (Table 4).

## 4. Discussion

To the best of our knowledge, this is the first study in Vietnam specifically designed to identify geriatric syndromes across all four key domains of the CGA—including physical health, functional ability, cognitive and mental health, and social-environmental circumstances—among older adults with sarcopenia. While previous studies in Vietnam have reported a high prevalence of sarcopenia and some related factors [4,6], none have systematically examined the distribution and burden of coexisting geriatric syndromes according to sarcopenia severity. Our findings therefore provide context-specific evidence from a rapidly ageing, middle-income setting and contribute to the growing international literature on sarcopenia and geriatric care.

Our findings provide novel insights into this relationship, highlighting the substantial burden of geriatric syndromes among older adults with sarcopenia, particularly those with severe sarcopenia. Notably, more than half (53.4%) of participants diagnosed with sarcopenia in our study met the criteria for severe sarcopenia. This proportion appears higher than population-based estimates from Asia, where severe sarcopenia prevalence is typically reported around 3–4%, and pooled estimates from global meta-analyses range from 2% to 9% [9,10]. Importantly, the 53.4% observed in our study represents the proportion of severe sarcopenia among individuals already diagnosed with sarcopenia, rather than a population prevalence. In addition, our study was conducted in a national tertiary geriatric hospital, where patients often present with greater functional impairment and clinical complexity. Across both non-severe and severe sarcopenia groups, the overall burden of geriatric syndromes was high. Importantly, the prevalence of these problems was consistently higher among individuals with severe sarcopenia, suggesting a higher overall burden of geriatric syndromes in this population. Gender-specific analyses further confirmed that participants with severe sarcopenia exhibited significantly lower handgrip strength, slower gait speed, and lower body mass index in both males and females, reinforcing the consistency and clinical relevance of these findings across sexes (Supplementary Table S1).

Associations between sarcopenia and conditions such as frailty, depression, sleep disturbance, and cognitive impairment have been described previously [13,14,15,16,17,18,19]. By confirming these associations in the Vietnamese context, our study supports the view that sarcopenia frequently coexists with multiple geriatric syndromes as part of a broader geriatric vulnerability profile. It is important to recognize that geriatric syndromes are highly prevalent in this population and should not be overlooked, as they frequently coexist and may interact to accelerate functional decline, increase healthcare utilization, and negatively impact quality of life [44,45]. Therefore, alongside interventions targeting nutrition and physical activity, the early recognition, systematic assessment, and proactive management of geriatric syndromes should be considered integral components of sarcopenia care. In settings such as Vietnam, where geriatric services and CGA are still developing, these findings may help guide clinical prioritization and inform resource planning for older adults with sarcopenia. These findings also highlight the importance of integrating multidimensional interventions into routine clinical care, including resistance exercise, nutritional optimization, and management of psychological and sleep-related conditions. Such approaches may help preserve functional independence and improve quality of life in older adults with sarcopenia, particularly in rapidly ageing and resource-constrained settings such as Vietnam.

The associations between depression, sleep disturbance and sarcopenia observed in our study are consistent with prior research and extend recent findings from Vietnamese older adults in whom sarcopenia was independently associated with depressive symptoms [46,47,48,49,50,51,52,53]. These relationships likely reflect shared behavioral and physiological mechanisms affecting both muscle function and psychological health [19,46,47,48,49,54,55,56,57,58,59,60,61,62,63,64]. The high prevalence of sleep disturbance observed in this study should be interpreted in the context of the PSQI threshold of ≥5, which is designed to identify clinically relevant poor sleep quality and is widely used in geriatric research. This high prevalence may also reflect the clinical complexity of older outpatients with sarcopenia, who often present with multiple comorbidities and functional impairments associated with sleep problems. Pathophysiological mechanisms such as insulin resistance, hypothalamic–pituitary–adrenal axis dysfunction, systemic inflammation, and hormonal changes may underlie this association [18]. Our findings extend this body of evidence by showing that these problems were more frequently observed among individuals with severe sarcopenia, suggesting that this group represents a clinically vulnerable population in whom routine screening for mood and sleep disorders may be warranted. In addition, several of the underlying mechanisms may be potentially modifiable, indicating that physical activity, nutritional status, and mood and sleep disturbances constitute relevant and clinically meaningful domains to consider in the care of individuals with severe sarcopenia.

In regression analyses, several associations observed in unadjusted and partially adjusted models—particularly for frailty, ADL and IADL impairment, cognitive impairment, and social isolation—were attenuated after full adjustment for physical activity and nutritional status. This pattern suggests that the relationship between sarcopenia severity and these geriatric syndromes may be partially mediated through reduced physical activity and suboptimal nutritional status. These factors are closely linked to muscle function as well as broader functional and psychosocial vulnerability, and may therefore represent intermediate pathways rather than simple confounders. Nonetheless, sarcopenia and frailty are known to be closely related but distinct entities that frequently coexist and share common risk factors [65,66,67,68,69,70,71]. Interventions targeting muscle strength and physical performance may have beneficial effects on functional outcomes relevant to both conditions [72]. In contrast, the strong association observed between severe sarcopenia and mobility impairment in the fully adjusted model should be interpreted with caution. Because severe sarcopenia is partly defined by low gait speed and mobility impairment was assessed using the Timed Up and Go test, both measures capture closely related domains of physical performance and may reflect overlapping constructs rather than a fully independent geriatric syndrome.

Taken together, our findings support approaching sarcopenia within a broader geriatric framework rather than as an isolated muscle disorder. The accumulation of multiple syndromes among individuals with severe sarcopenia highlights the need for comprehensive assessment and integrated management strategies, particularly in resource-constrained and rapidly ageing settings. Future prospective and interventional studies are needed to determine whether structured approaches such as CGA lead to improved functional outcomes, reduced complications, and better quality of life in individuals with sarcopenia, and whether assessment strategies should be adapted according to disease severity.

The relationships between sarcopenia severity and geriatric syndromes are likely complex and bidirectional. In addition to environmental and lifestyle factors, genetic susceptibility may also influence the development and progression of sarcopenia, contributing to inter-individual variability in vulnerability and clinical presentation. Several conditions examined in this study—including depression, malnutrition, low physical activity, and mobility limitation—may both contribute to and be associated with greater sarcopenia severity. For example, reduced physical activity and poor nutritional status may accelerate muscle loss, while sarcopenia may also be associated with reduced functional capacity and psychological well-being. Because of the cross-sectional design, the observed associations should be interpreted as correlational rather than causal, and longitudinal studies are needed to clarify temporal relationships. Future studies should also explore sex-specific predictors of reduced muscle function, physical activity, and geriatric syndromes to better inform targeted prevention and intervention strategies.

Sarcopenia remains a relatively under-recognized condition in Vietnam. To the best of our knowledge, this is the first study in the country to examine sarcopenia in parallel with a wide spectrum of geriatric syndromes spanning multiple domains. A notable strength of this study is the use of face-to-face assessments performed by trained clinicians, employing validated and feasible instruments within a comprehensive geriatric assessment framework. This standardized approach enabled a systematic and multidimensional evaluation of geriatric problems and minimized the risk of measurement bias. However, several limitations should be acknowledged. First, because this study was conducted in a tertiary geriatric referral hospital that receives patients from Hanoi and multiple provinces across northern Vietnam, participants may represent a more clinically complex population than community-dwelling older adults. Therefore, the findings may not be fully generalizable to all older adults with sarcopenia in Vietnam, particularly those in community or primary care settings. Second, as a cross-sectional study, causal relationships between sarcopenia severity and geriatric syndromes cannot be inferred. Future longitudinal, multicenter studies are needed to clarify temporal relationships, explore potential mechanisms, and evaluate whether comprehensive assessment and management strategies translate into measurable clinical benefits in this high-risk population. In addition, there is conceptual and measurement overlap between severe sarcopenia and certain geriatric syndromes, particularly mobility impairment, ADL impairment, IADL impairment, and frailty, which may have contributed to stronger observed associations. These findings should therefore be interpreted with appropriate caution. Furthermore, because multiple geriatric syndromes were examined, the possibility of chance findings due to multiple comparisons should be considered and the results should be interpreted in terms of overall patterns and clinical coherence rather than individual statistically significant associations. In addition, some covariates included in the fully adjusted models, particularly physical activity and nutritional status, may represent both confounders and potential mediators, which could have attenuated some observed associations. Third, this study did not include a non-sarcopenic comparison group, and we were therefore unable to directly compare the prevalence of geriatric syndromes between individuals with and without sarcopenia. Our study was designed specifically to examine differences according to sarcopenia severity among those already diagnosed with the condition. Future studies including both sarcopenic and non-sarcopenic populations would help clarify the full spectrum of associations.

## 5. Conclusions

Older Vietnamese adults with sarcopenia had a high prevalence of coexisting geriatric syndromes, with a greater burden observed in those with severe disease. Severe sarcopenia was independently associated with sleep disturbance, depression, and impaired mobility. These findings highlight clinically relevant domains that should be systematically evaluated within comprehensive geriatric assessment and integrated into multidisciplinary care.

## Figures and Tables

**Table 1:** Comprehensive Geriatric Assessment Components.

CGA Components	Tools	Methods	Assessment Criteria
Physical assessment			
Comorbidities	Charlson comorbidity index	Medical record review and Clinical examination	
Polypharmacy		Interview/ Prescription/ Medical record	≥5 different medications on a daily basis [27,28]
Nutrition	Mini Nutritional Assessment Short Form (MNA-SF) [29]	Interview	0–7 scores: malnourished 8–11: risk of malnutrition 12–14: normal
Hearing	Whisper test [30]	Clinical examination	Yes/no
Vision	Snellen test [31]	Clinical examination	Yes/no
Urinary incontinence	3 Incontinence Questionnaires [32]	Interview	Yes/no
Constipation	ROME IV criteria [33]	Interview	Yes/no
Frailty syndrome	Clinical Frailty Scale (CFS) [34]	Interview	CFS ≥ 4: frailty or clinically significant vulnerability
Falls in the last 12 months		Interview	Yes/no
Functional assessment			
Physical function	Activity Daily Living scale (ADL) [35]	Interview	<6 scores: ADL impairment (Scores range from 0 to 6)
	Instrumental Activity Daily Living scale (IADL) [36]	Interview	<8 scores: IADL impairment (Scores range from 0 to 8)
Mobility	Timed Up and Go test (TUG) [37]	Physical test	≥13.5 s: mobility impairment
Neuro-Psychological assessment			
Cognitive function	Mini-Mental State Examination (MMSE) [38]	Interview	≥24: normal <24: cognitive impairment
Depression	Geriatric Depression Scale 15-item (GDS 15) [39,40]	Interview	≥5 points: depression (Scores range from 0 to 15)
Sleep quality	Pittsburgh Sleep Quality Index (PSQI) [41,42]	Interview	≥5 points: Sleep disorder (Scores range from 0 to 27)
Social and environmental factor			
Social isolation	Lubben social network scale 6 items (LSNS-6) [43]	Interview	<12 scores: Social isolation (Scores range from 0 to 30)

**Table 2:** Characteristics of the study population by sarcopenia severity.

**Characteristic**	**Non-Severe** **Sarcopenia**	**Severe** **Sarcopenia**	***p*-Value**
	(n = 338, 46.6%)	(n = 388, 53.4%)	
Age, years (mean ± SD)	72.2 ± 7.7	76.3 ± 7.9	<0.001
Age groups, n (%)			
60–69	142 (42.0)	77 (19.9)	
70–79	134 (39.7)	158 (40.7)	<0.001
≥80	62 (18.3)	153 (39.4)	
Females, n (%)	271 (80.2)	291 (75.0)	0.10
Low education level, n (%)	165 (48.8)	192 (49.5)	0.86
(less than high school)			
Living with spouse/family, n (%)	321 (95.0)	366 (94.3)	0.70
Living in rural area, n (%)	159 (47.0)	161 (41.5)	0.13
Hospitalizations in the past 12 months, n (%)			
≥1 time	118 (34.9)	171 (44.1)	0.012
Chronic comorbidities, n (%)			
Hypertension	156 (46.2)	218 (56.2)	0.007
Diabetes	85 (25.1)	136 (35.1)	0.004
Heart failure	24 (7.1)	56 (14.4)	0.002
Stroke	15 (4.5)	25 (6.4)	0.24
Chronic ischemic heart disease	20 (5.9)	32 (8.2)	0.23
Lipid disorder	98 (29.1)	107 (27.6)	0.65
COPD	10 (3.0)	12 (3.1)	0.92
Knee osteoarthritis	71 (21.1)	92 (23.7)	0.39
Parkinson	35 (10.4)	51 (13.1)	0.25
Physical activity levels, n (%)			
Low	176 (52.5)	292 (76.4)	
Moderate	147 (43.9)	89 (23.3)	<0.001
High	12 (3.6)	1 (0.3)	
Mean gait speed, m/s, (mean ± SD)	0.71 ± 0.24	0.59 ± 0.20	<0.001
Mean SMI, kg/m^2^, (mean ± SD)	5.56 ± 0.72	5.17 ± 0.78	<0.001
Mean HGS, kg, (mean ± SD)	16.27 ± 6.15	13.46 ± 5.33	<0.001

SMI: Skeletal Muscle Index (kg/m^2^); HGS: Handgrip strength (kg).

**Table 3:** Prevalence of geriatric syndromes according to sarcopenia severity.

**Components of CGA**	**Non-Severe** **Sarcopenia**	**Severe** **Sarcopenia**	***p*-Value**
	(n = 338, 46.6%)	(n = 388, 53.4%)	
Physical assessment			
Charlson comorbidity index, mean ± SD	1.3 ± 1.3	1.9 ± 1.5	<0.001
Polypharmacy, n (%)	142 (42.0)	192 (49.5)	0.04
Malnourished/risk of malnutrition, n (%)	168 (50.0)	268 (69.1)	<0.001
Hearing impairment, n (%)	144 (42.6)	179 (46.1)	0.34
Visual impairment, n (%)	171 (50.6)	216 (55.7)	0.17
Urinary incontinence, n (%)	46 (13.6)	80 (20.6)	0.013
Constipation, n (%)	66 (19.5)	96 (24.7)	0.09
Frailty syndrome, n (%)	185 (54.7)	277 (71.4)	<0.001
Falls in the past 12 months, n (%)	48 (14.2)	71 (18.3)	0.14
Functional assessment			
ADL impairment, n (%)	110 (32.5)	205 (52.8)	<0.001
IADL impairment, n (%)	133 (39.3)	226 (58.2)	<0.001
Mobility impairment, n (%)	73 (21.6)	203 (52.3)	<0.001
Psychological assessment			
Cognitive impairment, n (%)	62 (18.3)	115 (29.6)	<0.001
Sleep disturbance, n (%)	228 (67.5)	308 (79.4)	<0.001
Depression, n (%)	118 (34.9)	213 (54.9)	<0.001
Social and environmental factors			
Social isolation, n (%)	99 (29.3)	151 (38.9)	0.006
Number of geriatric syndromes	5.3 ± 3.5	7.2 ± 3.4	<0.001
0	19 (5.6)	4 (1.0)	<0.001
1−5	157 (46.5)	118 (30.4)	
6−10	143 (42.3)	200 (51.6)	
11−15	19 (5.6)	66 (17.0)	

**Table 4:** Association between severe sarcopenia and selected geriatric syndromes.

CGA Components	Severe Sarcopenia		
	Model 1 OR (95%CI)	Model 2 OR (95%CI)	Model 3 OR (95%CI)
Polypharmacy	1.36 (1.02–1.83) *	1.30 (0.96–1.77)	1.11 (0.79–1.56)
Hearing impairment	1.15 (0.86–1.54)	0.98 (0.72–1.34)	0.92 (0.66–1.27)
Visual impairment	1.22 (0.91–1.63)	1.11 (0.82–1.50)	1.05 (0.76–2.23)
Urinary incontinence	1.64 (1.11–2.44) *	1.33 (0.88–2.01)	1.10 (0.71–1.68)
Constipation	1.35 (0.95–1.92)	1.30 (0.90–1.87)	1.19 (0.81–1.75)
Frailty syndrome	2.06 (1.52–2.81) *	1.72 (1.25–2.37) *	1.14 (0.79–1.64)
Falls in the past 12 months	1.38 (0.93–2.07)	1.32 (0.87–1.99)	1.28 (0.83–1.98)
ADL impairment	2.31 (1.71–3.13) *	1.83 (1.33–2.51) *	1.32 (0.93–1.87)
IADL impairment	2.17 (1.61–2.92) *	1.60 (1.16–2.20) *	1.17 (0.83–1.66)
Mobility impairment	3.98 (2.87–5.52) *	3.40 (2.43–4.76) *	3.01 (2.12–4.27) *
Cognitive impairment	1.88 (1.32–2.66) *	1.50 (1.04–2.17) *	1.18 (0.80–1.76)
Sleep disturbance	1.86 (1.33–2.60) *	1.90 (1.34–2.69) *	1.49 (1.02–2.18) *
Depression	2.27 (1.68–3.06) *	2.44 (1.78–3.34) *	1.90 (1.36–2.66) *
Social isolation	1.53 (1.12–2.09) *	1.49 (1.08–2.05) *	1.32 (0.94–1.95)

OR: odds ratio; CI: confidence interval. Model 1: Unadjusted. Model 2: Adjusted for age and gender. Model 3: Adjusted for age, gender, physical activity levels, malnutrition, hypertension, and diabetes. Goodness-of-fit for Model 3 (fully adjusted) was assessed using Nagelkerke R^2^ (range: 0.026–0.346), Hosmer-Lemeshow test (all *p* > 0.05), and the overall classification accuracy (range: 60.3–83.8%) (presented in Supplementary Table S2). * *p* < 0.05.

## Data Availability

The data presented in this study are available on request from the corresponding author. The data are not publicly available due to privacy and ethical restrictions.

## Supplementary Materials

The following supporting information can be downloaded at https://www.mdpi.com/article/10.3390/geriatrics11030051/s1: Table S1: Sex-specific comparisons of anthropometric, muscle function, and selected clinical characteristics. Table S2: Association between severe sarcopenia and comprehensive geriatric assessment components: fully adjusted logistic regression models and goodness-of-fit measures.

## Abbreviations

The following abbreviations are used in this manuscript:

ADL	Activity Daily Living
AWGS	Asian Working Group for Sarcopenia
CGA	Comprehensive Geriatric Assessment
GDS	Geriatric Depression Scale
HGS	Handgrip strength
IADL	Instrumental Activity Daily Living
IPAQ-SF	International Physical Activity Questionnaire short form
LSNS	Lubben social network scale
MNA-SF	Mini Nutritional Assessment Short Form
MMSE	Mini-Mental State Examination
PSQI	Pittsburgh Sleep Quality Index
SD	Standard Deviations
SMI	Skeletal Muscle Index
