# Supplementary material for: Sarcopenia Severity and the Accumulation of Geriatric Syndromes Among Older Adults: A Cross-Sectional Study from Vietnam
Source: Geriatrics (Basel). 2026 Apr 23;11(3):51. doi: 10.3390/geriatrics11030051 (PMC13214922; doi:10.3390/geriatrics11030051)
Supplement: Supplementary file 1 [file geriatrics-11-00051-s001.zip › geriatrics-4122158-supplementary.pdf]

Table S1: Sex-specific comparisons of anthropometric, muscle function, and selected clinical characteristics

Variable	Male			Female		
	Non-severe sarcopenia (n=67)	Severe sarcopenia (n=97)	p-value	Non-severe sarcopenia (n=271)	Severe sarcopenia (n=291)	p-value
**Anthropometric characteristics**						
Age, years	73.7±7.4	77.8±7.2	<0.001	71.8±7.7	75.8±8.0	<0.001
Height, cm	160.2±6.7	160.9±5.4	0.456	150.3±6.4	149.1±5.5	0.015
Weight, kg	56.0±8.0	53.5±6.8	0.030	50.3±7.1	46.9±7.0	<0.001
Body mass index, kg/m^2	21.8±2.6	20.7±2.7	0.009	22.3±2.9	21.1±2.9	<0.001
Body fat mass, kg	15.0±4.6	13.3±5.1	0.030	16.4±5.5	15.6±5.9	0.084
Percent Body Fat, %	26.6±6.2	24.8±7.1	0.095	32.1±7.4	31.9±8.3	0.703
**Muscle mass and function**						
Skeletal muscle mass index, kg/m^2	6.2±0.7	6.0±0.6	0.069	5.4±0.6	4.9±0.6	<0.001
Handgrip strength, kg	21.2±7.2	17.6±6.4	0.001	15.1±5.2	12.1±4.1	<0.001
Gait speed, m/s	0.7±0.2	0.6±0.2	0.005	0.7±0.2	0.6±0.2	<0.001
**Clinical characteristics**						
Charlson Comorbidity Index	2.0±1.3	2.2±1.5	0.415	1.2±1.2	1.7±1.5	<0.001
Physical activity levels, n (%)						
Low	37 (55.2)	74 (76.3)		142 (52.4)	220 (76.3)	
Moderate	28 (41.8)	23 (23.7)	0.008	119 (43.9)	68 (23.4)	<0.001
High	2 (3.0)	0 (0)		10 (3.7)	1 (0.3)	
Low educatinal level (less than high school)	26 (38.8)	31 (32.0)	0.365	138 (51.1)	161 (55.3)	0.317

Table S2: Association between severe sarcopenia and comprehensive geriatric assessment components: fully adjusted logistic regression models and goodness-of-fit measures

**CGA components**	**Model 3 OR (95%CI)**	**Goodness-of-fit measures**		
		Nagelkerke R^2	Hosmer-Lemeshow (p)	Classification Accuracy (%)
Polypharmacy	1.11 (0.79 – 1.56)	0.177	0.294	66.6
Hearing impairment	0.92 (0.66 – 1.27)	0.094	0.208	60.3
Visual impairment	1.05 (0.76 – 2.23)	0.081	0.830	61.2
Urinary incontinence	1.10 (0.71 – 1.68)	0.089	0.407	82.5
Constipation	1.19 (0.81 – 1.75)	0.033	0.082	77.8
Frailty syndrome	1.14 (0.79 – 1.64)	0.346	0.811	75.3
Falls in the past 12 months	1.28 (0.83 – 1.98)	0.026	0.882	83.8
ADL impairment	1.32 (0.93 – 1.87)	0.311	0.834	70.9
IADL impairment	1.17 (0.83 – 1.66)	0.305	0.139	72.2
Mobility impairment	3.01 (2.12 – 4.27)*	0.214	0.125	69.4
Cognitive impairment	1.18 (0.80 – 1.76)	0.216	0.341	76.4
Sleep disturbance	1.49 (1.02 – 2.18)*	0.169	0.372	75.3
Depression	1.90 (1.36 – 2.66)*	0.208	0.931	67.9
Social isolation	1.32 (0.94 – 1.95)	0.099	0.402	66.8

OR: odds ratio; CI: confidence interval.

Model 3: Fully adjusted models include age, sex, physical activity level, nutritional status, hypertension, and diabetes.

Model goodness-of-fit was assessed using Nagelkerke R^2, the Hosmer–Lemeshow test, and the overall classification accuracy.

Classification accuracy represents the percentage of correctly classified observations based on the logistic regression model.

A non-significant Hosmer–Lemeshow test indicates acceptable model fit.

* $p<0.05$.
